# Easier Said Than Done: Healthcare Professionals’ Barriers to the Provision of Patient-Centered Primary Care to Patients with Multimorbidity

**DOI:** 10.3390/ijerph18116057

**Published:** 2021-06-04

**Authors:** Sanne J. Kuipers, Anna P. Nieboer, Jane M. Cramm

**Affiliations:** Department of Socio-Medical Sciences, Erasmus School of Health Policy & Management, Erasmus University, 3062 PA Rotterdam, The Netherlands; nieboer@eshpm.eur.nl (A.P.N.); cramm@eshpm.eur.nl (J.M.C.)

**Keywords:** patient-centered care, multimorbidity, primary care, general practice, care delivery, semi-structured interview, qualitative study

## Abstract

Patient-centered care (PCC) has the potential to entail tailored primary care delivery according to the needs of patients with multimorbidity (two or more co-existing chronic conditions). To make primary care for these patients more patient centered, insight on healthcare professionals’ perceived PCC implementation barriers is needed. In this study, healthcare professionals’ perceived barriers to primary PCC delivery to patients with multimorbidity were investigated using a constructivist qualitative design based on semi-structured interviews with nine general and nurse practitioners from seven general practices in the Netherlands. Purposive sampling was used, and the interview content was analyzed to generate themes representing experienced barriers. Barriers were identified in all eight PCC dimensions (patient preferences, information and education, access to care, physical comfort, emotional support, family and friends, continuity and transition, and coordination of care). They include difficulties achieving mutual understanding between patients and healthcare professionals, professionals’ lack of training and education in new skills, data protection laws that impede adequate documentation and information sharing, time pressure, and conflicting financial incentives. These barriers pose true challenges to effective, sustainable PCC implementation at the patient, organizational, and national levels. Further improvement of primary care delivery to patients with multimorbidity is needed to overcome these barriers.

## 1. Introduction

Patient (or person)-centered care (PCC) receives a great deal of attention and has been adopted widely in healthcare organizations throughout the world [1,2,3,4,5,6]. In the past two decades, many interventions have been implemented to make healthcare organizations more patient centered. Commonly implemented PCC interventions for patients entail patient empowerment, physical support, and information provision; those for healthcare professionals focus mainly on education and training and improvement of the continuity and coordination of care [6].

With such efforts, most organizations claim to be patient centered; the reality, however, is more nuanced [7,8,9]. In theory, PCC should be delivered using a comprehensive approach, with multiple interventions tailored specifically to the needs of the most vulnerable groups in society (e.g., patients with less education, migration backgrounds, or low health literacy) [2]; in practice, achieving this goal remains a huge struggle [10,11,12]. This nuanced picture of PCC in practice is especially relevant for primary care delivery to patients with multimorbidity (two or more co-existing chronic conditions [13]), who are often considered to form one of the most vulnerable groups in society [14]. Globally, more than half of people aged >65 years have multiple chronic conditions, which are treated mainly in the primary care setting [15,16]. Patients with multimorbidity are often older, with lower socioeconomic status and fewer health literacy skills [16]. Multimorbidity is also more prevalent among patients with migration backgrounds than among those without migration backgrounds [17]. Furthermore, multimorbidity is often related to adverse patient outcomes, such as poor health, low quality of life, functional impairment, and a greater risk of mortality [15,18,19,20].

**Figure 1:**
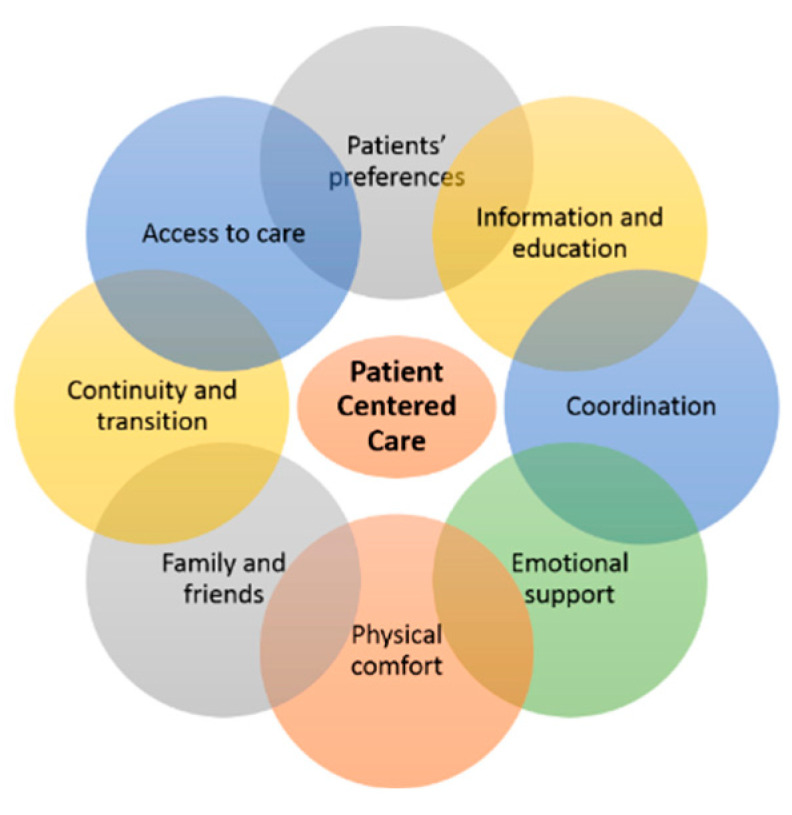
Framework of the eight dimensions of patient-centered care, as defined by the Picker Institute [24].

Current primary care delivery is not optimally tailored to the needs of patients with multimorbidity; PCC has the potential to overcome this obstacle [21,22,23]. The Picker Institute developed an eight-dimension framework that describes all aspects of PCC [24] (Figure 1): (1) patient preferences, (2) information and education, (3) access to care, (4) physical comfort, (5) emotional support, (6) family and friends, (7) coordination of care, and (8) continuity and transition. 

According to this framework, PCC delivery to patients with multimorbidity requires, among other efforts, that healthcare professionals strive to support patients in the setting and achievement of treatment goals guided by *patient preferences*. Patients with multi-morbidity can be viewed as being experts on their diseases [25] who should be empowered by healthcare professionals to be in charge of their own care. To do so, healthcare professionals should provide *information and education* that is accessible and understandable to all, regardless of education, age, educational background, or health literacy. Furthermore, PCC emphasizes the need for good *access to care*, meaning, among other characteristics, affordability and the accessibility of buildings to all patients, including those with mobility limitations. Moreover, as having many chronic conditions is often accompanied by physical problems, and as the perceived quality of the physical comfort (e.g., spatial layout) offered in healthcare settings affects the perceived quality of care, attention should be paid to patients’ *physical comfort* (e.g., management of sleeping problems, pain, shortness of breath; provision of comfortable facilities) [26]. Having multiple chronic conditions impacts patients’ lives, social relations, and/or jobs, and is often accompanied by feelings of anxiety and depression [27,28]. Thus, to be patient centered, healthcare professionals should offer *emotional support* to patients. Furthermore, chronic illnesses affect not only patients, but also their *family and friends* [29]. With PCC, healthcare professionals should involve these individuals in the care process, as they also have roles in care delivery and support [30]. Finally, care delivery to patients with multimorbidity often involves multiple healthcare professionals, within organizations (*coordination of care*) and across healthcare disciplines (*continuity and transition*). To ensure PCC, all healthcare professionals involved in care delivery to a multimorbid patient should be well informed, which involves regular and adequate transfer of information, and care delivery should be aligned to avoid fragmentation [31,32].

In practice, the Picker Institute’s framework [24] is often used for the development of PCC guidelines and interventions. An example of such interventions is the establishment of patient-centered medical homes, which serves as a model for high-quality primary care that is considered to be more effective than standard care for patients with chronic conditions [33]. A systematic review has shown that the organization of care according to these eight dimensions of PCC results in better organizational and patient outcomes [2].

Although a clear vision of PCC for patients with multimorbidity has been developed [34], PCC implementation in practice is not always straightforward. Barriers occasionally hamper adequate PCC delivery or prevent PCC implementation entirely. Healthcare professionals in management positions frequently mention the lack of time and funding as obstacles [23]. Multimorbid patients often have complex problems and needs, which take much time and effort to identify [35]. The identification of the problems at hand and the care and support required is particularly difficult for patients with low health literacy and/or education levels [36,37]. In addition, patients with multimorbidity form a heterogenous population requiring more than one type of PCC delivery [38]. Furthermore, most healthcare systems remain single disease oriented, and thus not adequately responsive to the needs of patients with multiple chronic conditions [39], resulting in complications in practice [40]. This situation reflects the need for and added value of PCC, as well as the challenges faced in its implementation. Despite agreement about the importance of PCC for patients with multimorbidity in the primary care setting, the realization of PCC in practice remains difficult. Although healthcare professionals’ perspectives of primary care delivery for patients with multimorbidity have been investigated [40,41,42,43], evidence from healthcare professionals regarding the sources of difficulties with PCC implementation for these patients is scarce. Thus, the identification of barriers to such implementation is a first step toward further improvement in practice.

### Study Aim

To make primary care for patients with multimorbidity more patient centered, insight on perceived barriers to PCC delivery for this population is needed. Thus, the present study was conducted to investigate such barriers, as perceived by healthcare professionals in a primary care setting.

## 2. Materials and Methods

### 2.1. Study Design

This study was conducted using a constructivist qualitative research design [44]. Data from semi-structured interviews were analyzed to identify barriers to PCC delivery for patients with multimorbidity in the primary care setting, as perceived by healthcare professionals (general practitioners (GPs) and nurse practitioners (NPs)). Its methodology is described according to the consolidated criteria for reporting a qualitative research checklist (e.g., participant selection, setting, data collection, analysis) [45].

### 2.2. Setting and Participants

All participating healthcare professionals, from seven GP practices in Noord-Brabant, the Netherlands, participated in a 1-year-long (2017–2018) PCC improvement program initiated by a regional cooperative of GPs (Zorggroep RCH Midden Brabant BV). The program’s aim was to improve primary PCC delivery to patients with multimorbidity. Participants attended meetings for the improvement of their knowledge about PCC and the sharing of their experiences with PCC implementation in practice. A toolbox of interventions for PCC improvement was provided, and participants were instructed in its use in several workshops (the PCC improvement program and intervention toolbox have been described in detail previously [34]). The first and third authors (SK and JC) were present at all program meetings.

At the end of the program, interviews were conducted to identify perceived barriers to PCC delivery for patients with multimorbidity in the primary care setting. This approach is similar to that used in previous qualitative studies of barriers to primary care delivery [46,47]. Sampling was purposive, with the intent of interviewing at least one GP and one NP per practice. The practices selected healthcare professionals for participation. As three practices had the same healthcare team, 10 interviews were planned. One interview was cancelled due to the participant’s illness. Thus, nine healthcare professionals (four GPs and five NPs; one male and eight females), comprising 43% of PCC improvement program participants, agreed to participate and were interviewed. After these interviews, the authors presented the themes to the healthcare professionals, and together, the group decided that no additional interview was needed, as all themes were recognized and no additional theme emerged. For the same reason, no repeat interview was conducted.

### 2.3. Ethics

The medical ethics committee of Erasmus Medical Centre, Rotterdam, the Netherlands, determined that the rules stipulated in the Medical Research Involving Human Subjects Act did not apply to this study (protocol no. MEC-2018-021). 

### 2.4. Data Collection

In January and February 2018, the first author conducted semi-structured interviews lasting about 1 h each. Each interview was conducted at the GP practice of the interviewee, with only the researcher and participant present. All interviewees were familiar with the purpose of the research and with the interviewer, with whom they had established relationships during prior program meetings. During the interviews, the eight PCC dimensions were used as a guide for consistency. Open questions (without a predetermined set of questions) were used to investigate the interviewees’ conceptualizations of each dimension of PCC, and of what could be further improved. Verbal informed consent was obtained from all participants. With the participants’ permission, the interviews were recorded digitally. No fieldnotes were made during the interviews.

### 2.5. Analysis

**Figure 2:**
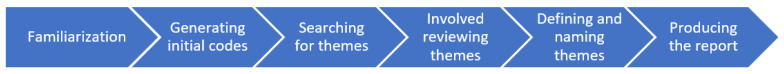
Six steps of thematic analysis according to Braun and Clarke [47].

Reflexive thematic analysis was applied to the data, based on the steps defined by Braun and Clarke [48], to identify patterns of meaning across the dataset. The authors analyzed the data inductively; coding and theme development were directed by its content. To identify patterns of meaning, six steps were defined for the analysis (Figure 2). First, all interviews were transcribed verbatim (~3.5 h per transcript), and the first author read the full transcripts to familiarize herself with the data. The respondents did not read the transcripts. Second, the first author coded the content using ATLAS.ti, (version 8.4.18; ATLAS.ti Scientific Software Development GmbH, Berlin, Germany). Third, all authors examined the codes and identified themes in each PCC dimension representing barriers to PCC delivery for patients with multimorbidity in the primary care setting identified by the respondents. Fourth, all authors reviewed and refined the themes, discussing their scope and names until agreement was reached (triangulation). Finally, to validate the findings, all themes were discussed during a meeting, with all 22 healthcare professionals participating in the PCC improvement program; the professionals recognized the themes raised, and no additional theme emerged during this meeting.

## 3. Results

Descriptive statistics of the participants are presented in Table 1. 

The healthcare professionals identified barriers (themes) in all eight PCC dimensions (Table 2). The barriers are presented by dimension, but described below in no specific order, as all of the dimensions are important for the improvement of PCC.

### 3.1. Patient Preferences

#### 3.1.1. Taking on a Coaching Role Takes Time and Calls for Additional Skills

The consideration of patients’ preferences, wishes, and needs in care delivery often requires a shift from paternalistic consulting toward a coaching role for healthcare professionals. According to the interviewees, this shift is not always easy. The assumption of this new role, and the exploration of patients’ preferences, take time.

*I have been working as a practitioner for many years and I have my ways, so I also have to get used to a change and a new approach to healthcare delivery.*

*(NP1)*

Moreover, this shift requires additional communication skills and techniques to enable healthcare professionals to explore patients’ preferences and support them in goalsetting. Not all healthcare professionals, however, have been trained or acquired these new skills, which makes PCC delivery challenging. Furthermore, not all healthcare professionals are willing to make this change.

*I still get very easily into sending mode. Sometimes you just convey certain information without having properly tested where the patient’s needs lie.*

*(GP1)*

#### 3.1.2. The Need for Mutual Understanding of Patients’ Needs

For adequate PCC delivery, a mutual understanding of patients’ needs and priorities is crucial; the interviewees reported that achieving such understanding can be challenging. For example, the exploration of patient needs and preferences is more difficult when there is a language barrier or cultural difference.

*Sometimes a language barrier or culture also makes it difficult. With a language barrier, patients do not always understand what is going on and that they have a say too. And culture also often does determine how people cope with their disease process. Often, they are used to me telling them what is wrong, what they have to do, and then they do it.*

*(GP4)*

#### 3.1.3. Not All Patients Want to Be Actively Involved

The exploration of patients’ wishes and needs is also more difficult when patients do not want to be actively involved in care delivery. Some patients have difficulties being proactive, sharing their perspectives, or setting goals concerning their care. They prefer care as usual, with goalsetting done mainly by their healthcare professionals. The receipt of care as usual can be considered as a patient preference, although healthcare professionals sometimes struggle with this factor.

*It can also be that the patient comes to me with very different expectations and does not feel the need to express what he wants, but adopts more of a consuming attitude: “well, just tell me how the blood sugar is and whether the blood pressure is okay and I will be satisfied.” Then it is difficult to find out what people really want with their health.*

*(GP1)*

### 3.2. Access to Care

#### 3.2.1. Agreements with Healthcare Insurers Do Not Fully Support PCC

The interviewees emphasized that the time needed to deliver PCC, especially to patients with multimorbidity, should not be underestimated. As NPs often have flexible consultation times, this barrier applies mainly to GPs. Most consultations with GPs last 10–20 min, which is a short period of time for patients with complex care needs. Blood pressure or glucose measurement and/or the discussion of other physical complaints often take up most of this time. Financial arrangements with healthcare insurers have restricted consultation durations, limiting multimorbid patients’ access to care. Spending more time with patients than agreed upon with health insurers is not rewarded. These agreements are thus perceived as barriers to PCC delivery, as the time pressure means that healthcare professionals cannot always discuss patients’ care preferences or set goals with them.

*What I find very strange is that if you tailor your care to the needs of the patient, help and invest in them well, then you get penalized very badly financially for that.*

*(GP2)*

*If I have only ten minutes, I go much less deeply than if I have double the time. Then I can ask a lot more thoroughly what the patient means and list all the options. Sure, I always try to do that, but really teaching the patients to make and set their own goals goes a bit further than that.*

*(GP4)*

Another example is the healthcare insurers’ predetermination of the number of follow-up visits for multimorbid patients. With PCC, this number should be determined according to patients’ preferences, but this is currently difficult, as the insurers take the performance of fewer follow-up visits to represent low-quality care delivery and do not provide reimbursement for visits beyond the number agreed upon.

*Well, if the patient says “I like it so much here I will come back next week,” you also have a problem. Because then he comes next week and the week after that, but you only get paid for two or three contacts a year. And that, of course, averages out. The health insurance company only looks at the care that was delivered. And if you get paid twice and you see him ten times, they would rather see that, than if you get paid three times and you only see him once.*

*(GP1)*

#### 3.2.2. Community Support Is Not Always (Financially) Accessible for Patients

Healthcare professionals often use community support elements, such as taxi rides to GP appointments for patients with mobility limitations, as part of good PCC provision. However, these services are not always (financially) accessible for patients, as they are often not reimbursed.

*Exercise programs can make a huge contribution to care. But people do not get reimbursed for it, and there is still a group of people with small budgets who cannot afford it themselves. In order to provide PCC, sometimes a bit of professional guidance to get and stay in motion is also very much needed. I think that is a real gap in the regulations.*

*(NP1)*

### 3.3. Physical Comfort

#### Struggles with the Offering of Physical Comfort at GP Practices

The interviewees acknowledged the importance of offering physical comfort at GP practices, but noted that they struggle with what to provide and what is considered to be sufficient (i.e., what exactly is “comfortable”). Moreover, they sometimes have limited options for comfort provision. For example, space limitations can make the provision of adequate privacy via separate waiting rooms and a separate front desk difficult. Furthermore, some interviewees expressed awareness that physical comfort (e.g., swinging doors) was suboptimal at their practices, but had no concrete plan to solve this problem.

*My consultation room is upstairs where you can only get to by stairs. That is not ideal for some patients. But the lack of space forces me to do this. Sometimes when people cannot manage it, I make house calls and some of the people we know about we try to schedule them for a day when we have a free consultation room downstairs. But this is becoming increasingly difficult because we are indeed short of space. I realize that we also have swing doors as a front door, which is not very handy with the wheelchair.*

*(NP5)*

### 3.4. Family and Friends

#### 3.4.1. Unfamiliarity with the Involvement of Family Members and Friends in Regular Consultations

The interviewees stated that they struggle with the involvement of multimorbid patients’ family members and friends in care delivery, including consultations, because they are simply not used to doing so. In addition, not all interviewees were aware of the benefits of this practice in terms of patient outcomes.

*Well we can always do better, but I do not know how. Then you have to learn yourself to bring up those kinds of things [private situations] more often. But I do not quite see how to do that in an ordinary consultation. I only do that in exceptional cases. I do not ask the standard diabetic patient how things are at home. I will bring it up, but not every three months, I think.*

*(NP2)*

#### 3.4.2. Consultation Time Is often Too Limited for the Involvement of Family Members and Friends

The GPs and NPs also stated that they often do not involve patients’ relatives due to the time required to do so and to pay attention to and address their needs and questions. As their consultation times for this patient population are often limited, GPs choose to pay more attention to other aspects of care delivery.

*The time is too limited. And if there is a problem, you would like to do something with it. And wanting to do something with it means the more things you bring up, the more problems there are, the more time you need to find a solution for all those problems.*

*(GP1)*

#### 3.4.3. Contradicting Needs and Wishes of Patients and Their Family Members and Friends

The interviewees explained that family members’ preferences sometimes contradict those of patients, which contributes to the difficulty of involving relatives in care delivery.

*Involving family is sometimes difficult. Sometimes I do get phone calls from [patients’] children. Sometimes that is nice, sometimes it is not. If several children are involved in the care delivery, and all want something different, it sometimes creates difficult situations.*

*(NP5)*

### 3.5. Emotional Support

#### 3.5.1. Patients Visit GP Practices Due to Physical, Rather Than Emotional, Problems

The interviewees recognized that not all patients think that their GP practice is the place to discuss emotional issues or the impacts of chronic diseases on one’s private life. Although some patients know that the exploration of such issues is the task of mental-health NPs, they do not believe it to be the task of GPs. This perspective may impede the provision of adequate emotional support to patients who need it.

*Sometimes you also see that there is some doubt if they [patients] can say it here, because how will we [healthcare professionals] think of it [an emotional problem].*

*(GP1)*

#### 3.5.2. Healthcare Professionals Do Not Always Address Emotional Problems

The interviewees acknowledged that they do not always address possible emotional problems accompanying multimorbidity.

*Of course, I do not always ask about it [emotional problems]. Yes, if people start talking about it themselves, I do listen. I do my best with that, or I suggest the accessible mental healthcare nurse practitioner. But there is not always attention to emotional aspects. Someone with diabetes with good values is doing well. Then I am not going to actively ask whether he is also under stress.*

*(NP2)*

Furthermore, not all interviewees felt comfortable discussing emotional aspects accompanying patients’ diseases, such as depressive feelings or anxiety.

*Well, there will undoubtedly be intrinsic factors in myself as well, on account of which I may be more likely to discuss certain things rather than other topics. I also bring my own person into a conversation. So that can be a barrier.*

*(NP1)*

#### 3.5.3. Healthcare Professionals Feel That It Is Not Their Task to Provide Emotional Support, and That Time Is Limited

The GPs interviewed also noted the lack of clear boundaries for the provision of emotional support, whether the recognition of problems is sufficient or more is needed. This factor is related to time pressure; the interviewees stated that they do not want patients to believe that they can make appointments solely to discuss emotional problems, as they feel that this is not their role and that time is limited.

*I do not have time myself to talk for half an hour every week, but the mental healthcare nurse practitioner does. Some people do like that, other people say no I do not want that, I just want to talk about it here. And then I think, no way I am going to free up my schedule to talk for half an hour every week. We also have to set boundaries.*

*(GP2)*

*If a patient is very sad, you cannot say “well, the time is up.” You do not do that. So yes, that also makes the planning of the consultation hours difficult, because they come for something and if everything else comes along, which is quite often, then it runs late. And you cannot schedule everyone for half an hour, because even if you were to work twenty-four hours a day, you still would not have seen all the patients. So, you always have to choose and share. And that is just annoying. You can never do the best for everyone and that is very frustrating.*

*(GP2)*

### 3.6. Information and Education

#### 3.6.1. Information Does Not Always Match the Situations of Multimorbid Patients

The interviewees emphasized the importance and difficulty of providing information specific to multimorbidity, as disease-specific information on comorbidities does not always exist.

*I would like to give more psycho-education, so people get more specific information. But that is difficult to do for such a wide range of conditions. There are so many things that play a role in multimorbidity.*

*(GP4)*

#### 3.6.2. Variation in Patients’ Health Literacy Makes the Alignment of Information and Education Difficult

Not only healthcare professionals, but also patients, need to possess skills to explore their preferences. Patients need to have health literacy and communication skills to share preferences and information and set goals. Thus, the interviewees found the lack of such skills to impede PCC delivery.

*You will see that patients with multimorbidity are often older people. And older people often look up to the doctor as well. And have a little less knowledge, they think, of all kinds of diseases, while of course that is not the case. Because they have been on Earth much longer than I have. But the elderly are more sensitive to it. The younger people can decide much easier, and often find a lot of information on the internet to make a targeted choice.*

*(GP1)*

The interviewees noted that health literacy skills vary greatly in this patient population, making the adjustment of information provision to individual patients difficult. It can be difficult to recognize what patients need to gain better health literacy skills, and to determine whether patients have truly understood the information provided.

*And as to low literacy, here in the village it is not too bad, but for someone who barely finished secondary school or did not finish it at all, it is obviously quite difficult to think about conditions, pills, solutions and options, to make a choice. And then it seems as if you have to be smart to make a good choice, but someone who is less educated can do that just as well. Provided that the information fits well. And of course, there is a barrier in that. Because as professionals we communicate on a completely different level. We use much more complicated words and terms that do not always come across.*

*(GP1)*

The interviewees mentioned that the development and use of multiple resources (e.g., brochures) adapted to all education levels and language backgrounds would aid the provision of good information aligned with patients’ needs and characteristics. Although such materials exist, the interviewees did not use them often.

*I could perhaps do more with the foreign people here in the district in terms of informational material. Because I do that a lot in Dutch now. Of course, they are often accompanied by someone who can speak Dutch, but then it all goes through an intermediary. And I think there are enough materials in other languages as well that are not yet available at the thuisarts website [which provides disease-specific information to patients].*

*(NP2)*

### 3.7. Coordination of Care

#### 3.7.1. Larger Numbers of Team Members Add Complexity to the Coordination of Care

According to the interviewees, adequate PCC delivery requires all practice team members to believe in the added value of this approach. They noted that the coordination of care differs between small and large teams in GP practices. For PCC, the same team should be involved in every instance of care delivery to a patient. However, coordination becomes more complex with the addition of team members (e.g., multiple assistants at the front desk, part-time workers).

*We were looking at how to divide the patients among three nurse practitioners. At first we had one nurse practitioner, and then of course there was nothing to divide. But now we have more. And one works only so many hours part time and the other works only so many hours part time. So, it all just has to fit, but coordinating this can be quite a challenge.*

*(GP1)*

*For a patient, it is quite difficult. Having your own general practitioner and a nurse practitioner is manageable. But there are also eight assistants they have to deal with, and I think that can be confusing. That could be organized better.*

*(NP1)*

#### 3.7.2. The Team Atmosphere Is Crucial for Improvement in an Organization

The interviewees emphasized the importance of the team’s morale and atmosphere for the adoption of a new approach. When no safe environment to provide feedback and ask critical questions exists, improvement is difficult.

*It is enjoyable to watch each other’s work and you can get a lot of tips and find many improvements by doing so. But feedback is sometimes given in such a way that makes it come across as hurtful or threatening. There must also be a sense of safety.*

*(GP1)*

### 3.8. Continuity and Transition

#### 3.8.1. A Longer Care Chain Entails Risks

In many cases, healthcare professionals from diverse disciplines in various healthcare settings (e.g., primary, hospital, community, and social care) are involved in care delivery to patients with multimorbidity. The interviewees noted that this situation may hinder the continuity of care; longer chains of care are more vulnerable to disruption.

*Because there are many healthcare settings involved, there are many links and each link is vulnerable. If I verbally pass something on to you and you pass it on to someone else and they pass it on to their colleague. After ten people, look what finally emerges.*

*(GP1)*

To ensure the continuity of care, collaboration among healthcare settings is very important. The GPs interviewed stated that they tried to take leading roles in managing the continuity and transition of care, but emphasized that this was easier said than done. The part-time work schedules of many healthcare professionals render the continuity of care even more difficult, due to difficulties with the scheduling of meetings and alignment of advice. Furthermore, the interviewees stated that they did not always know the expertise of professionals in other disciplines, especially those outside of the healthcare setting (i.e., in the community or social domain), which makes the transition of information and referral difficult.

*I think that as a GP I have a particular task when people see several specialists and those specialists are not always well informed about each other’s goals and treatments. Patients sometimes lose their way because of this, because they feel that there is not enough holistic collaboration. My job is to call or consult with the specialist or refer someone who is a bit older to a geriatrician. And then I sometimes ask specifically whether the geriatrician could take over the check-ups from the various specialists. But that is often not the case. If someone is a very specific rheumatologist or a patient has a cardiac or pulmonary condition, you do not let those specialists go easily. Then you sometimes have to call more often to get things coordinated. I think that takes a lot of energy. And it takes a lot of energy from the patient as well.*

*(GP3)*

*More and more people work part time. So, in any case you also get more and more people within the chain who are not always available at the time that you work.*

*(GP1)*

#### 3.8.2. Data Protection Laws Impede Adequate Documentation and Information Sharing

The interviewees identified data protection laws as barriers to PCC, and in particular to the continuity and transition of care. Good, complete documentation shared among all healthcare professionals involved in a patient’s care is important, but these laws prohibit the sharing of some information with professionals in all disciplines, resulting in the loss of (relevant) information. Medical information may be transferred only between medical doctors, and cannot be shared with paramedics, who are members of multidisciplinary teams providing PCC. The laws also make information sharing during multidisciplinary team meetings difficult.

*We have a pharmacy here in the building. I am not allowed to just hand over a list to the pharmacy saying these are all the people with heart failure, could you please check if the medication is okay. Because that is a data leak. So, I have to ask permission from each individual patient to tell the pharmacy that they have heart failure. And then if the patient says yes, then it is allowed. Otherwise it is not. So, you have to take a lot of steps to get there.*

*(GP2)*

*We are only allowed to transfer information to another physician. So, not all the allied healthcare professionals are allowed to have certain information, because that is all protected. We also have a chain information system, but everyone’s information is open to a limited extent. Most healthcare professionals involved really only get the referral and no additional information is allowed.*

*(GP2)*

The data protection laws also complicate communication with healthcare professionals involved in a patient’s care, as the (unprotected) exchange of emails is not permitted. This situation often results in a loss of efficiency in seeking to achieve continuity of care.

*Email traffic in primary care really needs to be implemented safely at breakneck speed, although it is apparently very difficult. This is really a shortcoming. This would allow us to communicate even better with the patient. For me as a NP, the GP is ultimately responsible, so I have to regularly consult with the GP and then call the patient back. The patient also has to stay at home especially for that phone call. With an email you can save a lot of time, but it will also help the patient since he can read everything back at leisure. If you start with medication, the patient has to pick it up at the pharmacy, take it at a certain time for a certain amount of time. That is a lot of information, and putting that in an email might be more convenient.*

*(NP3)*

#### 3.8.3. Information and Communications Technology Systems Are Not Optimally Designed to Ensure Care Continuity and Transition

According to the interviewees, the data information systems used within the organization and for the entire care chain are not optimally designed to function concurrently. Given the use of two different systems, not all relevant information is transferred adequately to all professionals on multidisciplinary care teams. This situation complicates communication among all healthcare providers involved and may result in the fragmentation of care.

*When I report on diabetes care, all the doctors involved can just see it in the chain information system [CIS]. But within the practice we work with a GP information system (GIS), but those two systems do not always work well together. For example, when patients last visited the optometrist. Nine times out of ten, the data is correctly processed in CIS but sometimes it does not come across well in GIS. So, for example, they go to their GP for an annual check-up and the GP asks when was the last time they saw the optometrist? Sometimes the patient cannot remember, so the GP looks in GIS and cannot find the report. Then they have to ask me to look in CIS to look it up. This is not very efficient.*

*(NP5)*

## 4. Discussion

This study was performed to investigate barriers to PCC delivery to patients with multimorbidity, as perceived by healthcare professionals in a primary care setting. Although the participating healthcare professionals acknowledged the value of PCC in this context, they identified barriers in all eight PCC dimensions. 

### 4.1. Patient Preferences

According to the study findings, healthcare professionals face difficulties in making the shift from a paternalistic consulting to a coaching role; the assumption of a new role takes time, and additional skills are necessary to, for example, thoroughly explore patient preferences. Such changes of mindset have been mentioned frequently as barriers to PCC implementation [49]. Furthermore, although patient-centered communication encompasses several skills, such as the expression of empathy and shared decision making [50], many healthcare professionals are not trained in such skills and do not realize that their possession could help them improve their patient-centered communication [50,51]. Communication training could achieve this goal [52], potentially enabling healthcare professionals to gain a better understanding of their patients’ conditions and care needs, in turn resulting in better treatment alignment [53]. Healthcare professionals also encounter barriers with regard to patient preferences (e.g., language barriers) when creating mutual understanding with their patients. Language barriers perceived by patients and healthcare professionals have been found to impede PCC delivery to immigrant and refugee women [10]. 

In addition, healthcare professionals who participated in this study reported feeling that not all patients want to be actively involved in their care and/or have difficulties with goalsetting. Patients have been found to differ in their proactivity and skills for active PCC involvement [38]. Although a patient’s preference for care as usual should be respected, we emphasize the need for thorough examination of whether the patient truly does not want to be in charge of his or her care, or whether the selection of care as usual is simply easier for him or her, as he or she may have difficulties with expressing his or her needs or preferences. The latter reflects the need for extra support from healthcare professionals to identify patients’ needs and preferences.

### 4.2. Access to Care

In the *access to care* dimension, healthcare professionals reported the lack of reimbursement for care provided as a barrier to effective PCC implementation. PCC often requires that healthcare professionals spend more time and exert more effort during consultations and in additional training sessions and workshops, and that they collaborate with professionals in other healthcare disciplines. The lack of financial structures supporting such activities may hamper the sustainability and widespread embedding of PCC into care systems in the long term. Concerns similar to those identified in this study have been raised by many healthcare professionals participating in programs aiming to improve the quality of primary care (e.g., integrated primary care for community-dwelling frail older persons, interventions based on the chronic care model) [54,55]. Supporting financial structures are often described as prerequisites for the effective and sustainable implementation of healthcare delivery [56,57]. In addition, as the financial resources of patients with multimorbidity vary [35], the creation of supportive financial structures also accounts for community support that may be inaccessible to patients with fewer resources.

### 4.3. Physical Comfort

The healthcare professionals reported that they struggled with how to provide physical comfort in their GP practices. A systematic review revealed differences in preferences regarding essential aspects of physical comfort provided in healthcare organizations among departments and occupants [58]. Additional research is needed to identify specific aspects of physical comfort preferred by patients with multimorbidity. 

### 4.4. Family and Friends

The study participants reported several barriers in this dimension. They acknowledged that they had difficulty involving patients’ relatives in care delivery because they are simply not used to doing so, and not all healthcare professionals were aware of the benefits of doing so. Patients with chronic diseases have been found to involve their family members and friends more often when their care needs become too complex to self-manage and when worse health outcomes become more likely [59,60]. The study participants also reported that their consultation time is too limited to incorporate all aspects of PCC. As patients with multimorbidity often have physical complaints, most of the professionals’ attention is devoted to these problems, leaving limited time to address relatives’ needs and questions [40,61]. Finally, the healthcare professionals experienced difficulties when they faced contradicting needs of patients and their family members. In another study, patient–family disagreements also were identified as a barrier to family involvement in primary care [62]. 

### 4.5. Emotional Support

This study revealed that patients with multimorbidity do not think their GPs’ tasks include the discussion of emotional aspects of their conditions, as has previous research (38). GPs likely feel the same, although a 2014 mental healthcare reform in the Netherlands designated emotional support as a GP task [63]. The aforementioned barrier that consultation time is often spent fully on the addressing of the physical aspects of patients’ conditions also applies to this dimension. However, as patients with multimorbidity often experience high emotional burdens related to their conditions, emotional support of these patients should receive more attention [27,28].

### 4.6. Information and Education

Healthcare professionals participating in this study emphasized the importance of patients’ possession of health literacy and communication skills, which allows them to participate in PCC delivery. The alignment of information provided with multimorbid patients’ needs and backgrounds has been shown to be important to increase patient-centeredness [34]. This study revealed wide variation in such literacy and skills among patients with multimorbidity. This is in accordance with the previous identification of subgroups of patients with multimorbidity based on personal resources such as communication and health literacy skills [35]. Moreover, health literacy skills are often considered to be fundamental for patients who want to be in charge of their care [64]. Previous research provides insight in how PCC delivery can be aligned to the (differences in) care needs of patients with multimorbidity [38]. Furthermore, this study revealed a barrier related to the provision of information to patients with multimorbidity, as most available information is disease specific. The same barrier was identified in a systematic review describing the challenges that GPs face in managing patients with multimorbidity [40].

### 4.7. Coordination of Care

According to the study participants, optimal PCC delivery requires that all healthcare professionals in an organization are motivated to achieve change and improvement, and that the environment is supportive. When not all such professionals are motivated or able to change, improvement may be difficult. Consequently, larger teams may add complexity to the achievement of improvement. According to Fleuren et al. [56], organizational size, colleagues’ support, and the extent to which the task orientation beliefs of healthcare professionals fit the innovation goals are important determinants for healthcare innovation. 

### 4.8. Continuity and Transition

The study participants reported three barriers in the *continuity and transition* dimension. They reported that adequate information sharing is difficult to achieve when working with large teams of healthcare professionals across multiple settings. A study investigating how GP practices should organize their care for patients with multimorbidity to increase patient-centeredness showed that multidisciplinary work is very important and can be strengthened by the organization of multidisciplinary meetings [34]. A systematic review showed that fragmentation between primary and secondary care poses a major challenge to the provision of care to patients with multimorbidity [40]. Second, the study participants reported that data protection laws restrict information sharing among healthcare professionals from multiple disciplines involved in individual patients’ care. Third, they emphasized that data information systems within organizations and for entire care chains are not optimally designed for concurrent functioning. Previous studies have revealed similar challenges to the continuity of care [65,66]. The inadequacy of information and communications technology systems may endanger the continuity of care, which is especially important for patients with multimorbidity, many of whom require multidisciplinary healthcare teams. Optimal technology and supportive laws are often described as prerequisites for the effective and sustainable implementation of healthcare delivery [56,57].

### 4.9. Practical Implications and Future Research

The barriers identified in this study pose true challenges in the effort to effectively and sustainably implement PCC at the patient, organizational, and national levels. At the patient level, most identified barriers were related to the variation in patients’ care needs and health literacy skills. These differences should be considered when developing care plans according to the PCC framework. At the organizational level, this study showed that not all healthcare professionals are aware of and/or trained in all elements of PCC delivery. Training and education of healthcare professionals should be initiated to increase their awareness and skills related to patient-centered communication, the involvement of patients’ family members and friends, and the discussion of patients’ emotional status, thereby improving care delivery to patients with multimorbidity. At the national level, challenges are related to data protection laws that restrict information sharing among healthcare settings, and to the lack of financial structures supporting PCC implementation; both of these factors are considered to be prerequisites for the effective and sustainable implementation of healthcare delivery [56,57]. Future research and policies should focus on meeting organizational preconditions to enable investment in preventive care across the lifespan and to make PCC the best way forward.

### 4.10. Limitations

Several limitations of this study should be considered when interpreting its results. First, the generalizability of the results may be limited, as this study was conducted with primary healthcare professionals in the Noord-Brabant region of the Netherlands. Future research should investigate the experiences of healthcare professionals with regard to barriers to PCC implementation in other regions, countries, and healthcare settings. Second, the sample of nine healthcare professionals may be considered to be small. However, this sample size is similar to those used in other qualitative health and well-being studies [67,68,69,70]. We selected it carefully, inviting 50% of all healthcare professionals from the GP practices participating in the PCC improvement program. Furthermore, the data are rich and were discussed during a meeting with all PCC program participants for validation; all healthcare professionals agreed with the findings, and no new theme was raised. 

## 5. Conclusions

PCC has the potential to entail the tailored delivery of primary care according to the needs of patients with multimorbidity. PCC implementation in practice, however, is often difficult due to the existence of barriers. At the patient, organizational, and national levels, barriers were identified in all eight dimensions of PCC (patient preferences, information and education, access to care, physical comfort, emotional support, family and friends, continuity and transition, and coordination of care) in this study. They include difficulties with the achievement of mutual understanding between patients and healthcare professionals, the lack of healthcare professionals’ training and education in new skills, data protection laws that impede adequate documentation and information sharing, time pressure, and conflicting financial incentives. These barriers pose true challenges to effective and sustainable PCC implementation for patients with multimorbidity. 

## Figures and Tables

**Table 1:** Descriptive statistics.

Participant	Gender	Age (Years)	Employment at Organization	Workhours/Week
GP 1	Male	52	≥10 years	≥36 h
GP 2	Female	42	≥10 years	≥36 h
GP 3	Female	53	5–10 years	≥36 h
GP 4	Female	37	3–5 years	29–36 h
NP 1	Female	57	≥10 years	≥36 h
NP 2	Female	37	3–5 years	≥36 h
NP 3	Female	38	≤1 year	≤16 h
NP 4	Female	61	≥10 years	≥36 h
NP 5	Female	46	3–5 years	≥36 h
Overall (years/% of all participants)	89%	47	33.3% ≥10 years	56% ≥36 h

GP: General practitioner, NP: Nurse practitioner.

**Table 2:** Overview of barriers to patient-centered care (PCC) for patients with multimorbidity.

PCC Dimension	Barrier
Patient preferences	-Taking on a coaching role takes time and calls for additional skills
	-The need for mutual understanding of patients’ needs
	-Not all patients want to be actively involved
Access to care	-Agreements with healthcare insurers do not fully support PCC
	-Community support is not always (financially) accessible for patients
Physical comfort	-Struggles with the offering of physical comfort at GP practices
Family and friends	-Unfamiliarity with the involvement of family member and friends in regular consultations
	-Consultation time is often too limited for the involvement of family members and friends
	-Contradicting needs and wishes of patients and their family members and friends
Emotional support	-Patients visit GP practices due to physical, rather than emotional, problems
	-Healthcare professionals do not always address emotional problems
	-Healthcare professionals feel that it is not their task to provide emotional support, and that time is limited
Information and education	-Information does not always match the situation of multimorbid patients
	-Variation in patients’ health literacy makes the alignment of information and education difficult
Coordination of care	-Larger numbers of team members add complexity to the coordination of care
	-The team atmosphere is crucial for improvement in an organization
Continuity and transition	-A longer care chain entails risks
	-Data protection laws impede adequate documentation and information sharing
	-Information and communications technology systems are not optimally designed to ensure care continuity and transition

## Data Availability

The data are available upon (reasonable) request.
